# Supplementary material for: Interseismic strain build-up on the submarine North Anatolian Fault offshore Istanbul
Source: Nat Commun. 2019 Jul 8;10:3006. doi: 10.1038/s41467-019-11016-z (PMC6614505; doi:10.1038/s41467-019-11016-z)
Supplement: Supplementary file 1 — Supplementary Information PDF Format [file 41467_2019_11016_MOESM1_ESM.pdf]

Supplementary Information

Interseismic Strain Build-up on the Submarine North Anatolian Fault Offshore Istanbul

Lange et al.

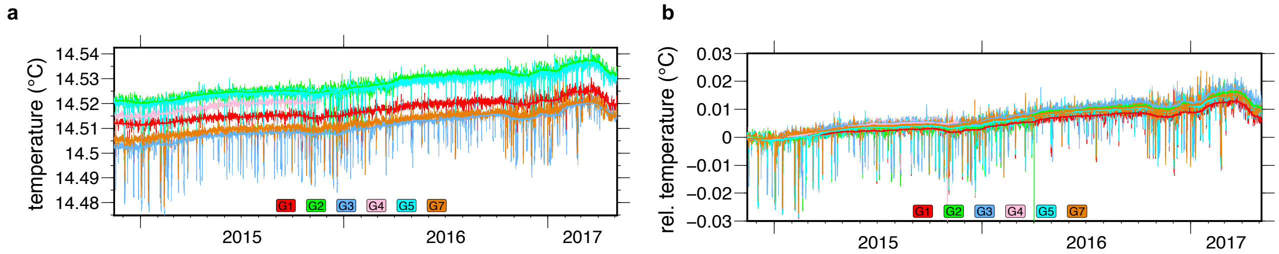

Supplementary Figure 1. High-resolution temperature data used for the baseline calculation. a Absolute temperatures for all stations show an increase in temperature of approx. 0.007 °C yr^{-1}. Temperature sensors of the French network had a long time drift and were not used for the baseline calculation. Therefore, the time-series of the close-by G-station temperature sensors were interpolated onto the measurement times of the F-stations. F-station temperature values are not shown since they would overlay and obscure the G-data. Station G4 stopped logging temperature after 19 November 2015. **b** Relative temperature data used for the baseline calculation. For better visualization values were set to zero at the beginning of the experiment. Black lines indicate monthly means.

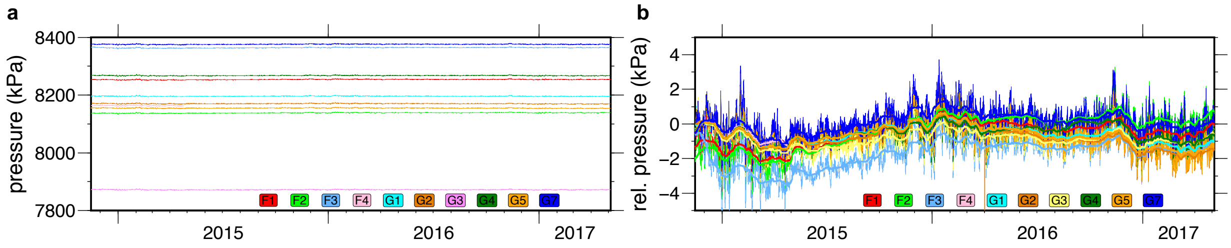

Supplementary Figure 2. Pressure data. a Absolute pressure for all stations. **b** Relative pressure changes for all stations. For better visualization values were set to zero at the beginning of the experiment. Lines indicate monthly means. F2 measured a relative increase of approximately 1 kPa (equivalent to 10 cm water column change) relative to closeby station G5 which we interpret as most likely due to a systematic pressure drift of station F2. In general, the pressure sensors used are known to have a long term mean drift of 0.88+/-0.73 kPa yr^{-1} [1]. As a result, drift and the differences between the pressure measurements might all be explained with sensor drift. Resolution of pressure is around 10 Pa corresponding to an effective depth resolution of ~1 mm.

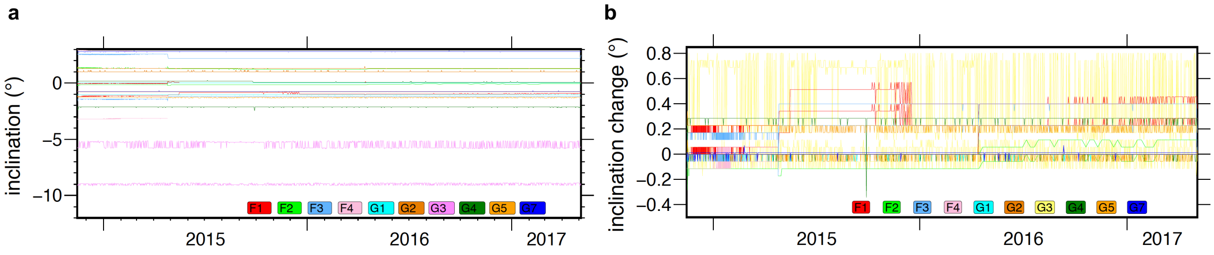

Supplementary Figure 3. Inclinometer data. a Absolute inclinometer data of all stations documenting the stability of the transponders. Since the orientation of the sensors is not known, pitch and roll only relate to the instrument reference frame. **b** Relative inclination change. For better visualization values were set to zero at the beginning of the experiment. Station G3 is located on an inclined slope and has the largest forward- and backward tilt of maximal 0.8°. Most stations have tilt smaller than the amplitude resolution of the inclinometer of 0.057° which equals to a maximal 4 mm lateral movement of the top of a 4 m high structure. Station G3 (shown with yellow lines) is located on a slope and tilted by 8° shows variations of inclination of 0.23° which equals to a maximal 1.6 cm lateral movement of a vertical 4 m high structure.

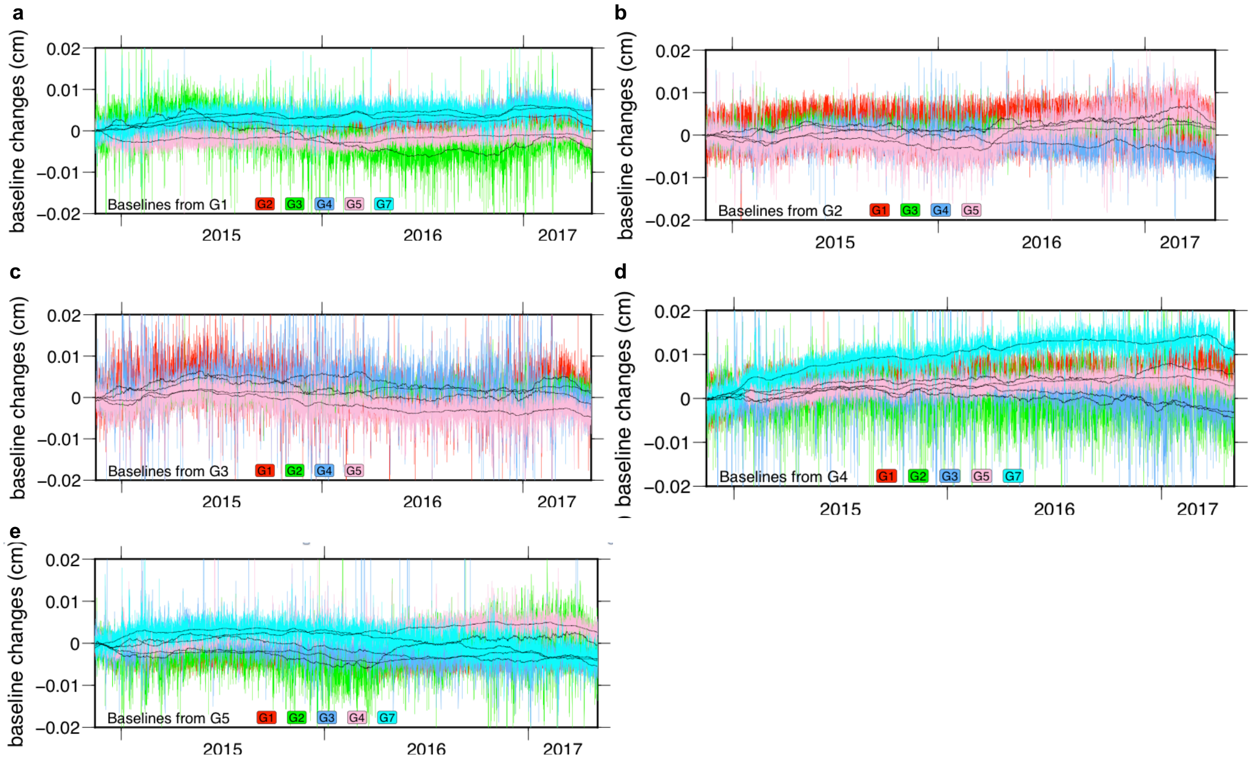

Supplementary Figure 4. Baselines from one G-transponder to all others. Black lines indicate 14-days average (median). Due to different baseline lengths, absolute values are difficult to compare. Therefore, zero is set arbitrarily at the beginning of the experiment. As discussed in the text, the baselines were linearly de-trended using a strain rate of $1.8x10^{-6}$ per year, corresponding to 1.8 mm baseline correction for a 1 km long baseline. Baselines of G4 after 19 November 2015 are based on the temperature measurements taken from F5 and are therefore not reliable (see discussion above).

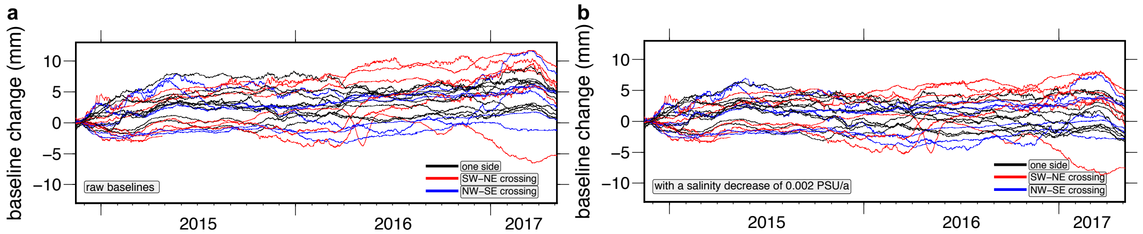

Supplementary Figure 5. Baseline length changes. **a** Baselines for all stations calculated using the sound speed estimated from pressure, temperature and a constant salinity. The average baseline lengthening of 3.6 mm is likely originating from a linear salinity decrease at a rate of -0.002 PSU yr^{-1} resulting in apparent lengthening of baselines. See the main text for discussion about the linear correction. **b** Same as panel a, but after removing the mean strain of 4.5^{-6} (corresponding to a baseline decrease of 4.5 mm for a 1000 m long baseline during the deployment time) estimated from all baselines on one side of the fault. The baselines are measured in the direction of the curved acoustic ray[2] traveling from one transponder to the other and therefore include the horizontal and vertical components of the length changes.

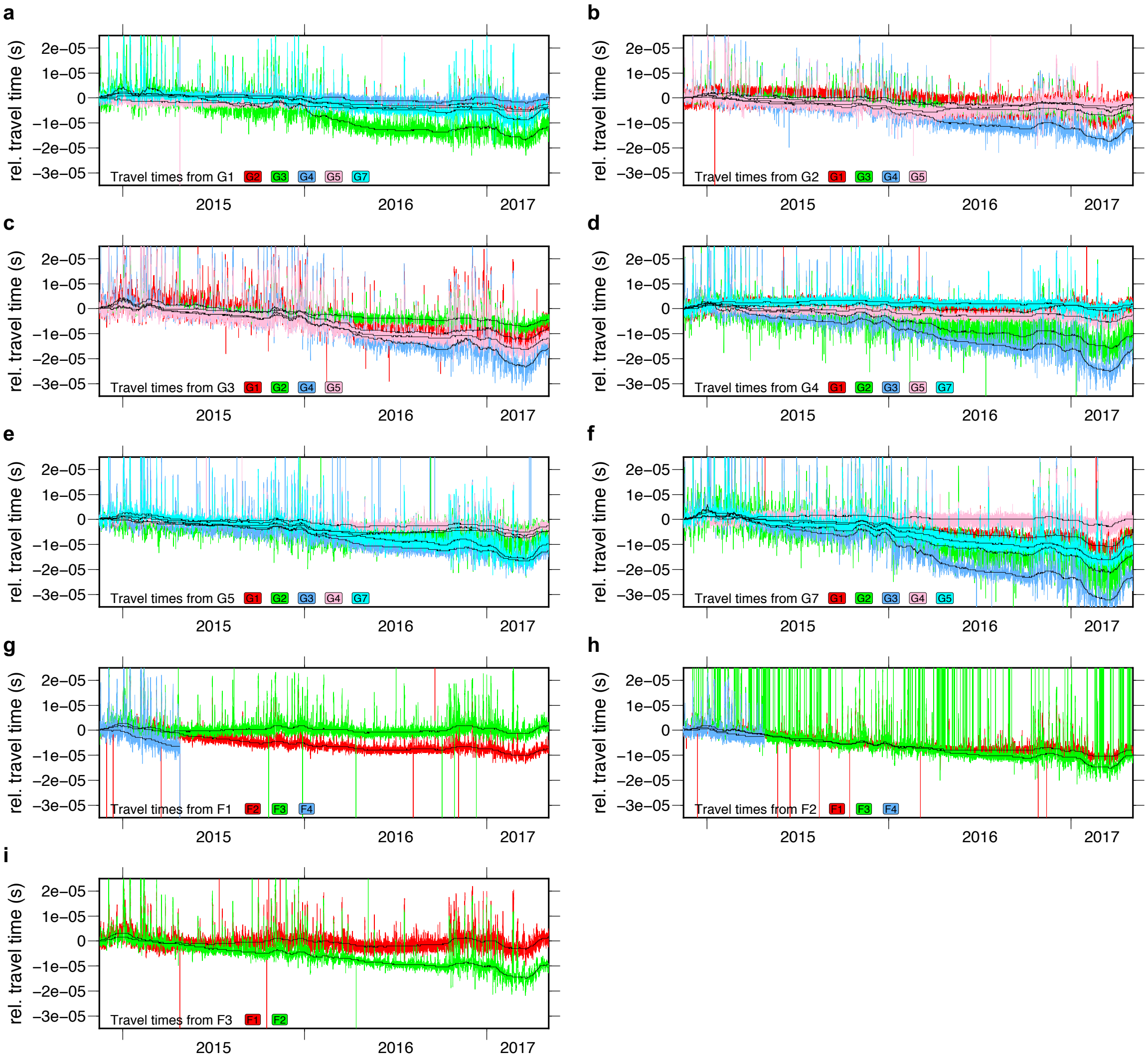

Supplementary Figure 6. Measured travel times. Relative travel-times for all baselines measured from one transponder to all others. Due to different baseline lengths, absolute values are difficult to compare. Therefore, zero is set arbitrarily at the beginning of the experiment. Transponder names are given in the bottom left.

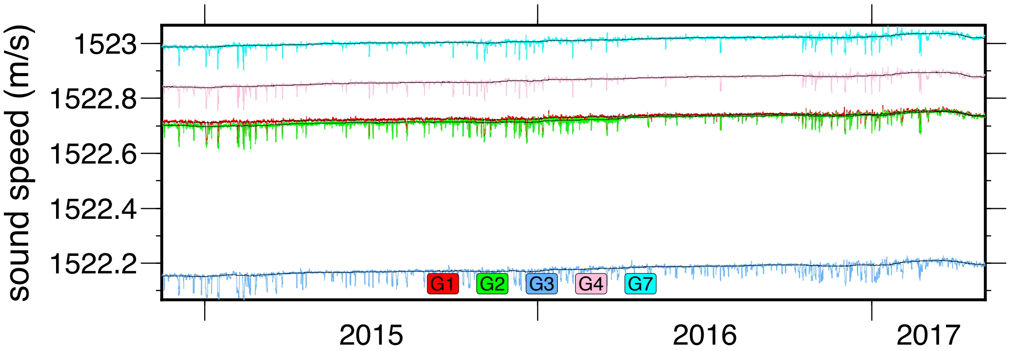

Supplementary Figure 7. Estimated sound speed. Sound speed of water calculated from pressure, temperature and a constant salinity for all G-stations. Sound speed differs between stations due to different water depths.

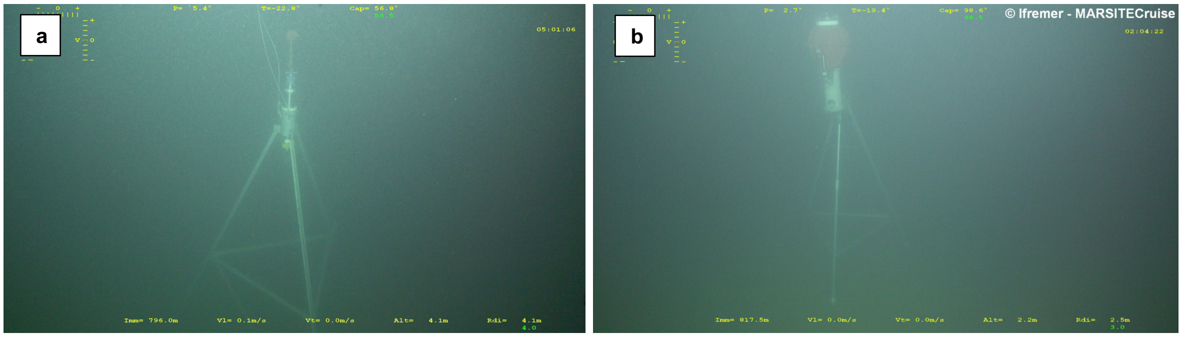

Supplementary Figure 8. Station on the seafloor. Remotely Operated Vehicle (ROV) images showing transponder G3 (panel a) and F4 (panel b) on the seafloor. The photos were taken during the MARSITE cruise of research vessel Pourquois Pas? shortly after the installation on 2 November 2014. Frames are approximately 4 m high and the total weight under water of the G-transponders is 311 kg to support stability of the installation.

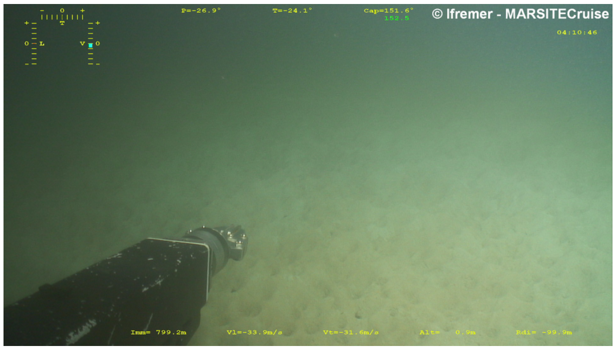

Supplementary Figure 9. Seafloor image. ROV manipulator arm touching the seafloor. The seafloor in the area of the geodetic deployment consists of muddy sediments.

baseline	baseline length at end of deployment	baseline inclination	baseline drift from linear regression (raw data)	drift uncertainty from linear regression (raw data)	drift uncertainty (raw data)	strain (raw data)	baseline change at end of deployment (raw data)	Baseline change corrected for salinity change (Fig. 2)	Basline change rate corrected for salinity change (Fig. 2)
ID-ID	(m)	(°)	(mm)	(mm yr^{-1})	(mm yr^{-1})	(dimensionless)	(mm)	(mm)	(mm yr^{-1})
F2-F3	870.44	-1.20	3.225	0.006	0.008	5.62698E-06	4.9	1.00	0.415
F2-F4	499.18	0.50	-1.001	0.014	0.013	-1.05303E-05	-5.3	-7.49	-3.123
F3-F2	870.44	1.20	2.900	0.008	0.008	4.41075E-06	3.8	-0.06	-0.026
G1-G2	500.06	0.40	2.369	0.010	0.010	9.69793E-06	4.8	2.61	1.087
G1-G3	977.23	1.70	1.882	0.025	0.025	8.07988E-06	7.9	3.52	1.465
G1-G5	363.65	0.10	0.257	0.010	0.004	-3.33919E-06	-1.2	-2.84	-1.185
G1-G7	887.29	-1.20	3.038	0.012	0.012	6.63526E-06	5.9	1.91	0.796
G2-G1	500.06	-0.40	2.161	0.009	0.009	9.09449E-06	4.5	2.31	0.961
G2-G3	497.16	3.00	2.276	0.008	0.007	1.01783E-05	5.1	2.83	1.180
G2-G5	515.15	-0.30	3.359	0.024	0.028	6.05517E-06	3.1	0.81	0.338
G2-G7	1329.92	-0.90	3.113	0.024	0.024	4.01819E-06	5.3	-0.62	-0.257
G3-G1	977.24	-1.70	-0.670	0.028	0.025	2.19627E-06	2.1	-2.23	-0.931
G3-G2	497.16	-3.00	1.980	0.007	0.007	9.34042E-06	4.6	2.42	1.006
G3-G5	855.00	-1.90	-0.007	0.014	0.012	6.20012E-07	0.5	-3.30	-1.376
G3-G7	1717.88	-1.60	0.749	0.022	0.022	2.82349E-06	4.9	-2.85	-1.188
G5-G1	363.64	-0.10	1.400	0.008	0.013	1.09166E-06	0.4	-1.23	-0.514
G5-G2	515.16	0.30	4.590	0.025	0.020	1.25827E-05	6.5	4.17	1.739
G5-G3	855.00	1.90	0.464	0.014	0.012	1.56283E-06	1.3	-2.50	-1.040
G5-G7	863.08	-1.30	-0.503	0.017	0.014	9.48331E-07	0.8	-3.05	-1.271
G7-G1	887.29	1.20	3.886	0.012	0.012	1.02315E-05	9.1	5.10	2.125
G7-G5	863.09	1.30	0.158	0.019	0.017	2.2792E-06	2.0	-1.90	-0.792
mean	771.67	-0.10	1.697	0.015	0.014	4.45733E-06	3.4	-0.07	-0.028

Supplementary Table 1. Details of estimated baselines, strains, and baseline drifts. Linear regression was done without the constant salinity decrease of 0.002 PSU/a.

depth below seafloor	Vp	Vs (km)	density	rigidity	geological unit
		Vs=(Vp-1.36)/1.16	*ρ=0.31(Vp/1000)$^{0.25}$*	*μ=ρ*Vs^2*	
(km)	(km s^{-1})	(km s^{-1})	(g/cm^{-3})	(GPa)	
0.00	1.46	0.09	1.92	0.01	
0.68	1.52	0.14	1.94	0.04	
0.81	2.10	0.64	2.10	0.85	
1.70	2.60	1.07	2.21	2.53	
3.08	4.16	2.41	2.49	14.51	pre-kinematic basement
3.08	4.50	2.71	2.54	18.60	
4.64	4.90	3.05	2.59	24.15	crystalline basement
4.64	5.67	3.72	2.69	37.14	
8.98	6.00	4.00	2.73	43.65	

Supplementary Table 2. Rigidity estimate of sediment below the geodetic network. We used the seismic P-wave velocity (v_p) profile and depth of geological units from seismic refraction observations located ~5 km south-west of the geodetic network[3]. Shear wave velocity (v_s) was estimated using the empirical mudrock line[4] from v_p velocities. Density was estimated using Gardner's empirical relation[5] from v_p and rigidity using the standard relation from density and v_s. Because the empirical relations include significant uncertainties we used for the modelling rigidity ratio of 4 between the upper (weak) and lower (strong) layer and modelled the weak layer with 3 km depth and 4.5 km depth, corresponding to the pre-kinematic basement and crystalline basement depth[3].

segment name	*shear modulus*	segment length	seismic moment (M0)	*moment magnitude (Mw)*
	(GPa)	*(km)*	*(Nm)*	*()*
Kumburgaz Basin	35	34	7.0661E+19	7.1995
Kumburgaz Basin	layered	34	5.6391E+19	7.1341
Çınarcik Basin	35	59	1.2366E+20	7.3615
Çınarcik Basin	layered	59	9.8686E+19	7.2962
Kumburgaz and Çınarcik Basin	35	93	1.9432E+20	7.4923
Kumburgaz and Çınarcik Basin	*layered*	93	1.5508E+20	7.4270

Supplementary Table 3. Magnitude estimates for different scenarios. Accumulated seismic moment and moment magnitude estimates for the Kumburgaz and Çınarcik Basin (Fig. 1). We estimated the moment magnitude for constant shear modulus of 35 GPa and for rocks with increasing shear modulus with depth using the values from Supplementary Table 2. The seismic moment[6] was calculated for an accumulated slip of 4m and a fault locking depth of 15 km and converted to equivalent moment magnitude[7]. For the horizontally layered shear modulus scenario the seismic moment is 25% smaller compared to a constant shear modulus of 35 GPa. The difference in moment magnitude is small (0.065 magnitude units) due to the logarithmic conversion from seismic moment to moment magnitude.

Supplementary References

1. Polster, A., Fabian, M. & Villinger, H. Effective resolution and drift of Paroscientific pressure sensors derived from long-term seafloor measurements. *Geochem. Geophys. Geosystems* **10**, (2009).
2. Petersen, F., Kopp, H., Lange, D., Hannemann, K. & Urlaub, M. Measuring tectonic seafloor deformation and strain-build up with acoustic direct-path ranging. *J. Geodyn.* **124**, 14-24 (2019).
3. Bécel, A. *et al.* North Marmara Trough architecture of basin infill, basement and faults, from PSDM reflection and OBS refraction seismics. *Tectonophysics* **490**, 1–14 (2010).
4. Castagna, J. P., Batzle, M. L. & Eastwood, R. L. Relationships between compressional-wave and shear-wave velocities in clastic silicate rocks. *Geophysics* **50**, 571–581 (1985).
5. Gardner, G. H. F., Gardner, L. W. & Gregory, A. R. Formation velocity and density; the diagnostic basics for stratigraphic traps. *Geophysics* **39**, 770–780 (1974).
6. Aki, K. Generation and propagation of G waves from the Niigata Earthquake of June 16, 1964. Part 2. Estimation of earthquake movement, released energy, and stress-strain drop from the G wave spectrum. *Bull Earthq Res Inst* **44**, 73–88 (1966).
7. Hanks, T. C. & Kanamori, H. A Moment Magnitude Scale. *J. Geophys. Res. Solid Earth* **84**, 2348–2350 (1979).
